# Cognitive and negative symptoms in schizophrenia with L‐Carnosine adjuvant therapy – A randomized double‐blind placebo‐controlled study

**DOI:** 10.1002/prp2.1074

**Published:** 2023-03-22

**Authors:** Hema Tharoor, Sindhu Maran, Antra K. Chandan, Manikandan Pari, Shruti Rao, Jothilakshmi Durairaj

**Affiliations:** ^1^ Schizophrenia Research Foundation Chennai Tamil Nadu India

**Keywords:** antioxidant, antipsychotics, executive function, nutraceutical, psychopathology, supplement

## Abstract

The antioxidant L‐Carnosine is reported to improve negative and cognitive symptoms in Schizophrenia. A randomized double‐blind placebo‐controlled study was planned to study the effectiveness of adjuvant L‐Carnosine therapy in patients with Schizophrenia. 100 eligible patients with predominant negative symptoms as measured by scale for assessment of negative symptoms (SANS total score ≥ 60) and Schizophrenia diagnosis (International Classification of Disorder‐Tenth Edition, ICD‐10) were recruited. They were randomly allocated to receive a fixed dose of either 400 mg L‐Carnosine or identical placebo for 3 months and increased to 800 mg from 13th week till completion of study. Primary outcome measures assessed changes in SANS scores with L‐Carnosine at 24 weeks compared to baseline, 4 and 12 weeks. Secondary outcome measures were done to assess the improvement in cognitive symptoms (executive function, attention, and memory) at 24 weeks using subtests of NIMHANS (National Institute for Mental Health and Neurosciences) cognitive battery. Side effects were assessed using adverse events reporting form. The attention scores (*p* = .023) showed significant differences in patients receiving 800 mg of L‐Carnosine at the end of the study. There were no significant differences in negative symptoms in the two arms at study completion. L‐Carnosine dosing of 800 mg may be a promising agent to enhance executive functions in Schizophrenia.

**Abbreviations**
ANOVA: analysis of variance
ASD: autism spectrum disorder
AVLT: auditory verbal learning test
CGI: clinical global impression
COVID‐19: Coronavirus disease
CTRI: Clinical Trial Registry‐India
ECG: electrocardiogram
GABA: gamma aminobutyric acid
LOCF: last observation carried forward
NIMHANS: National Institute for Mental Health and Neurosciences
NMDA: N‐methyl‐D‐aspartate receptor
RCT: randomized controlled trial
SANS: scale for assessment of negative symptoms
SAPS: scale for assessment of positive symptoms
SD: standard deviation
TRAIL–A: trail making tests A
TRAIL–B: trail making tests B

## 1 INTRODUCTION

L‐Carnosine is a naturally occurring dipeptide (composed of β‐alanine and L‐histidine) which is co‐localized and released with glutamate and is a widely consumed dietary supplement. β‐alanine has the ability to reverse the blocking actions of GABA (Gamma‐Aminobutyric acid) receptors^1^ and L‐histidine is involved in regulating food intake and the neurotransmitters serotonin, dopamine, and norepinephrine.^2^ Cognitive deficits are predominant in patients with schizophrenia.^3^ Dysregulation of N‐methyl‐D‐aspartate receptor (NMDAR) activity and glutamate are associated with negative and cognitive symptoms in Schizophrenia and treatment with L‐Carnosine is known to regulate NMDAR activity.^4^, ^5^, ^6^ Some observational studies suggest that lower β‐alanine and L‐histidine may be associated with schizophrenia and that treatment with antipsychotics reversed serum histidine and alanine levels.^7^ L‐Carnosine is also utilized for the synthesis of histamine, which is an important neuromodulator with a role in schizophrenia and mediated by histamine receptors.^8^ An early important milestone in L‐Carnosine supplementation (800 mg) in autism spectrum disorder (ASD) for 8 weeks showed decreased autism severity scores compared with placebo, especially in behavior, socialization, and communication sub‐scores.^9^ Two randomized controlled trials evaluated L‐Carnosine supplementation (2 g/day) with chronic schizophrenia on stable antipsychotic treatments and reported improvements in total scores as well as negative symptom sub‐scores for schizophrenia and improvement in several cognitive domains.^10^, ^11^ Therefore, this randomized controlled trial (RCT) was conceived with the objective of studying the effectiveness of improving negative and cognitive symptoms with adjuvant L‐Carnosine therapy in patients with schizophrenia.

## 2 METHODS AND MATERIALS

### 2.1 Study design and participants recruitment

An RCT complying with the standard protocol items recommendations for interventional trials (SPIRIT) statement was conducted.^12^ This was a 24 weeks, randomized, double‐blind, parallel‐group, placebo‐controlled study to examine the efficacy of add‐on L‐Carnosine in reducing negative and cognitive symptoms of schizophrenia. Patients with schizophrenia fulfilling the inclusion criteria were recruited from inpatient and outpatient departments of Schizophrenia Research Foundation, Chennai, India from June 2019 to December 2020. Written informed consent was obtained. The study was described to each participant with a witness (usually a caregiver) present. Institutional Ethics Committee clearance was obtained before the commencement of the study. A total of 120 eligible patients were approached, out of which 100 consented to participate in the study. Patients were randomized to receive either L‐Carnosine or placebo for 6 months. Study participants were assessed at baseline, 1 month, 3 months, and at the end of trial period (24 weeks from baseline). The trial was registered in the clinical trial registry in February 2019 (CTRI/2019/02/017646).

### 2.2 Randomization and blinding

A total of 100 participants were randomized to receive either L‐Carnosine or placebo after initial screening was completed and informed consent obtained. Allocation to treatment arm was randomly assigned in a 1:1 ratio by an independent researcher using random numbers generated. A computerized random number generator was used for the generation of randomization codes using permuted block randomization technique. The placebo tablet was not distinguishable in appearance (shape, size, color, and taste) from L‐Carnosine so as to conceal treatment allocation and blinding. Treating clinicians were also blinded to group allocation to ensure that trial treatment did not influence clinical care. All data, including biological samples, were kept in a secure location under appropriate storage conditions. The patients, rater, research assistant, laboratory assistants, and statisticians remained blinded throughout the entire trial. Table S1 depicts the time frame of enrollment, allocation, and assessments in SPIRIT format.^12^

### 2.3 Inclusion and exclusion criteria

The inclusion criteria were: (a) participants were required to meet International Classification of Disorder‐Tenth Edition criteria for schizophrenia with predominant negative symptoms as measured by SANS total scores ≥60 at baseline; (b) cognitive deficit below 15th percentile in any one cognitive domain measured using National Institute for Mental Health and Neurosciences (NIMHANS) Neuropsychological battery; and^13^ (c) age between 18 and 45 years.

Participants were excluded if they had (a) any other concurrent co‐morbid axis I diagnosis including substance use or dependence in the past 12 months; (b) history of serious medical/neurological disorder, known allergy to L‐carnosine; (c) past history of head injury resulting in loss of consciousness or neurosurgery; (d) mental retardation; (e) pregnant or lactating women; and (f) uncooperative due to severity of illness.

A sample size of 76 was calculated based on previous studies on augmentative therapy in stable schizophrenia. Considering an attrition of 20%, a sample size of 92 was considered which was then rounded off to 100.

### 2.4 Interventions

Patients were randomly allocated to receive either 400 mg daily of Carnosine or identical placebo for a period of 3 months. L‐Carnosine dose was titrated to 800 mg in the next 3 months. This dose was decided based on previously conducted study by Chez et al (2002). Besides, the maximum dose of L‐Carnosine approved for research in India is 800 mg. Weekly telephonic interviews were conducted by research staff to ensure adherence and also to monitor for adverse events. Antipsychotic and other prescribed psychotropic medications were continued (treatment as usual) during the entire study period. Participants were instructed not to take any other vitamins and carnosine supplements, while maintaining their regular diet and physical activity during the trial.

### 2.5 Clinical assessments

Sociodemographic data and relevant clinical and treatment variables were collected with the help of a semistructured proforma using medical records at baseline. Patients were assessed using scale for assessment of negative symptoms (SANS)^14^ and scale for assessment of positive symptoms (SAPS)^15^ at baseline, 4, 12, and 24 weeks. CGI ‐S (clinical global impression)^16^ was used to measure illness severity and CGI‐1 was administered at 4, 12, and 24 weeks. Selective items from the NIMHANS neuropsychological battery^13^ measured cognition at baseline and end of study (24 weeks). The cognitive domains assessed were executive function, attention, planning, and memory. The battery of tests consisted of 5 subtests of Trail making tests A and B (TRAIL A and TRAIL B), Digit Vigilance Test, Auditory Verbal Learning Test (AVLT), and the Tower of London.

### 2.6 Safety

Safety was evaluated using the adverse events reporting form^10^ which includes vital signs monitoring (temperature, blood pressure, pulse rate, height, weight, and waist circumference) electrocardiogram (ECG) and laboratory tests. Adverse events and vital signs monitoring were systematically evaluated at each visit (baseline, 4, 12, and 24 weeks) using a checklist. Blood samples were obtained by venipuncture, and assessed for complete blood count, random blood glucose, renal and liver function tests at baseline, 12 and 24 weeks. ECG was also taken at baseline, 12 and 24 weeks. Table S2 depicts the time frame for assessments of parameters included in the safety monitoring schedule.

### 2.7 Outcome measures

Changes in SANS and CGI scores at 24 weeks were the primary outcome measures. Secondary outcome measures included improvement in cognitive symptoms (executive function, attention, and memory) at 24 weeks assessed using subtests of NIMHANS battery.

### 2.8 Statistical analysis

Demographic data were analyzed with descriptive statistics such as mean with SD for the continuous and categorical variables. Frequencies and percentages have been reported. All analyses were based on the intention‐to‐treat sample and were performed using the last observation carried forward (LOCF) procedure. All continuous variables were compared across the groups using an independent *t* test. The dependent variables “SANS,” “SAPS,” and “CGI” were measured at 4‐time points: baseline, 1 month, 3 months, and 6 months. Mauchly's test for the sphericity assumption was violated for “SANS” and “SAPS” scores (*p* < .001). To overcome the issue of sphericity, Repeated Measure anova with Greenhouse–Geisser was performed. A *p* < .05 was considered statistically significant. Analysis of data was done using the Statistical Package for Social Sciences version 16.^17^

## 3 RESULTS

### 3.1 Baseline patient characteristics

**FIGURE 1:**
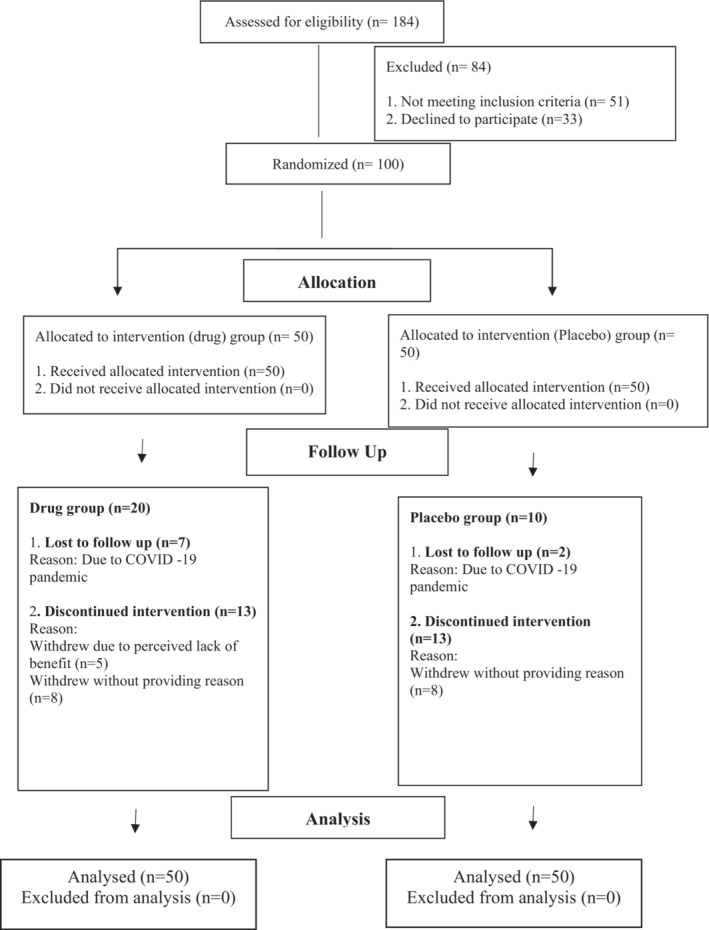
CONSORT flow diagram of the enrollment, allocation, follow‐up, and analysis.

Of the 100 recruited, 30 patients dropped out from the study. Of these, 11 withdrew before the first assessment and for 9 patients end line assessments could not be done due to COVID 19 lockdown restrictions. Hence, 70 patients completed the study as shown in Figure 1. There was no significant difference between drug and placebo groups in any of the sociodemographic and clinical variables as shown in Table S3. Baseline SANS, SAPS, CGI scores did not differ in the two groups (Table 1). No serious adverse events were reported in both the groups.

**TABLE 1:** Repeated measure analysis for comparison of the two treatment effects on SANS, SAPS, CGI scores.

Variables	Group		*p* value
	Drug (*n* = 50)	Placebo (*n* = 50)	
	Mean ± SD	Mean ± SD	
SANS			
Baseline	84.0 ± 18.4	80.2 ± 20.6	.834
1 month	78.82 ± 17.7	75.3 ± 23.0	
3 months	68.3 ± 20.5	63.8 ± 24.0	
6 months	57.3 ± 26.8	51.1 ± 28.0	
SAPS			
Baseline	28.3 ± 30.0	30.5 ± 30.0	.379
1 month	24.9 ± 26.7	26.7 ± 25.0	
3 months	24.2 ± 26.7	21.8 ± 24.2	
6 months	17.5 ± 22.1	20.0 ± 24.4	
CGI			
Baseline	3.7 ± 0.9	4.1 ± 0.8	.296
1 month	4.1 ± 2	4.7 ± 2.0	
3 months	5.4 ± 2.5	5.3 ± 2.4	
6 months	5.1 ± 2.1	4.7 ± 2.4	

Abbreviations: CGI, clinical global impression; *n*, number; SANS, Scale for Assessment for Negative symptoms; SAPS, Scale for Assessment for Positive Symptoms; SD, standard deviation.

### 3.2 Negative symptoms

The repeated measures anova was performed. Mauchly's test of sphericity indicated that the assumption of sphericity had been violated, *χ* ^2^ (2) = 71.9, *p* < .001, and therefore, a Greenhouse–Geisser correction was used. There is no significant difference in the SANS score between drug and placebo group (*F* (1.9195.9) = 0.18, *p* = .834) as shown in Table 1 at baseline, 4, 12, and 24 weeks. Individual domains of SANS scores did not differ in the drug and placebo group as shown in Table 2.

**TABLE 2:** Repeated measure analysis for comparison of the two treatment effects on SANS subscale domains.

Variables	Group		Test within subject effects F ratio with p value
	Drug (*N* = 50)	Placebo (*N* = 50)	
	Mean ± SD	Mean ± SD	
Affective flattening or blunting			
Baseline	26.0 ± 7.2	25.3 ± 8.8	*F* (1.633, 158.372) = 2.087, *p* = .137
1 month	24.7 ± 6.7	22.6 ± 7.9	
3 months	21.3 ± 6.8	18.8 ± 8.6	
6 months	17.7 ± 9.3	13.6 ± 10.1	
Alogia			
Baseline	14.6 ± 5.3	14.3 ± 5.8	*F* (1.858, 182.071) = 60.815, *p* = .639
1 month	14.4 ± 5.2	13.4 ± 5.6	
3 months	12.4 ± 5.2	11.3 ± 5.7	
6 months	9.6 ± 5.4	8.6 ± 6.6	
Avolition/apathy			
Baseline	13.3 ± 3.8	13.3 ± 3.9	*F* (2.081, 203.916) = 1.916, *p* = .148
1 month	12.7 ± 3.5	12.3 ± 5.2	
3 months	10.8 ± 3.5	9.0 ± 4.8	
6 months	8.3 ± 4.9	7.4 ± 5.2	
Anhedonia/asociality			
Baseline	19.4 ± 4.1	18.7 ± 5.4	*F* (1.672, 163.871) = 2.338, *p* = .109
1 month	18.9 ± 3.5	16.8 ± 5.6	
3 months	17.2 ± 4.4	14.6 ± 6.1	
6 months	14.8 ± 6.3	11.6 ± 6.7	
Attention			
Baseline	10.9 ± 2.8	11.0 ± 3.3	*F* (5.256 218.842) = 0.486, *p* = .638
1 month	10.5 ± 2.8	9.9 ± 3.0	
3 months	8.8 ± 3.3	8.7 ± 3.7	
6 months	7.1 ± 4.5	7.0 ± 3.8	

Abbreviations: *n*, number; SANS, scale for assessment for negative symptoms; SD, standard deviation.

### 3.3 Positive symptoms and CGI

Mauchly's test of sphericity indicated that the assumption of sphericity had been violated for SAPS and CGI scores and the chi‐square values are *χ* ^2^ (2) = 77.6, *p* < .001, *χ* ^2^ (2) = 37.1, *p* < .001, respectively, and therefore, a Greenhouse–Geisser correction was used. Both SAPS and CGI scores did not differ in the two groups (Table 1).

### 3.4 Cognitive symptoms

**FIGURE 2:**
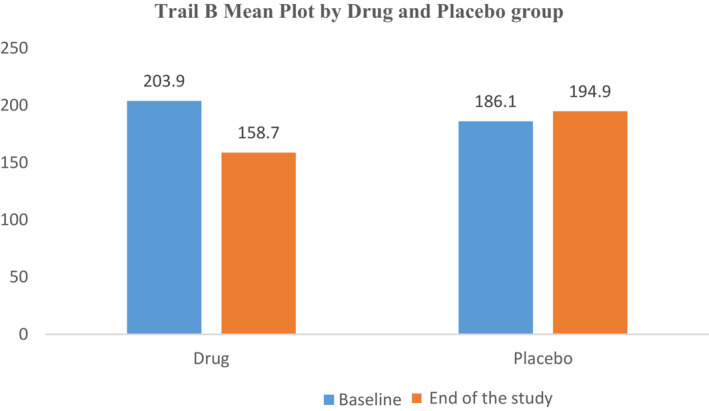
TRAIL B mean plot between drug and the placebo group.

The L‐Carnosine treated group showed a significantly greater improvement only in trail making tests B when compared to placebo group (*p* = .023) as shown in Table 3 and Figure 2. There are no significant gender differences in Trail A & B scores from baseline to end of study between the two groups. The ratio of Trail A divided by B scores also was not statistically significant between the genders.

**TABLE 3:** Comparison of cognitive domains between the two groups using *t* tests.

Variables	Group			
	Drug (*n* = 50)	Placebo (*n* = 50)	Mean difference (95% CI)	*p* value
	Mean ± SD	Mean ± SD		
TRAIL A				
Baseline	78.1 ± 36.8	76.3 ± 40.1	4.5 (−6.0 to 14.8)	.389
End of study	69.4 ± 29.6	71.7 ± 38.9		
TRAIL B				
Baseline	203.9 ± 135	186.1 ± 124.3	53.2 (7.4 to 99.4)	.023*
End of study	158.7 ± 92.1	194.9 ± 164.3		
Digit vigilance time				
Baseline	753.4 ± 328.7	655 ± 349.8	14.3 (−297.8 to 269.2)	.92
End of study	817.2 ± 962.4	711.3 ± 322.4		
Digit vigilance error				
Baseline	42.5 ± 68.3	37.9 ± 40.9	4.7 (−22.2 to 12.7)	.59
End of study	40.3 ± 57.5	29.6 ± 31.4		
AVLT(LTPR)				
Baseline	102.7 ± 123.7	73.6 ± 46.3	29.1 (−7.2 to 65.4)	.114
End of study	94 ± 43.2	96.7 ± 40.6		
TOL TNPMM				
Baseline	7.1 ± 2.3	8 ± 2.2	0.6 (−1.7 to 0.6)	.322
End of study	6.6 ± 2.9	6.8 ± 2.6		

Abbreviations: AVLT, auditory verbal learning test; LTPR, long term percent retention; TNPMM, total number of problems solved with minimum moves; TOL, tower of London.

* *p* < .05.

### 3.5 Adverse events

During the entire study, 22 adverse effects were recorded. The frequency of side effects did not differ significantly between two groups as shown in Table 4. No major adverse event was reported and they did not account for the dropouts.

**TABLE 4:** Adverse events in intervention and control group.

Side effects	L‐Carnosine *n* (%)	Placebo *n* (%)
Digestive		
Dry mouth, constipation, nausea	1 (5.9)	0 (0)
Metabolic		
Weight gain	0 (0)	1 (5.6)
Weight loss	0 (0)	1 (5.6)
Nervous		
Dizziness, sleepiness		
Slurred speech, lethargy	0 (0)	0 (0)
Dermatological		
Skin rash, dry skin, pruritis	0 (0)	0 (0)
Musculoskeletal:		
Foot and joint pain	0 (0)	0 (0)

## 4 DISCUSSION

L‐Carnosine is widely used as an antioxidant and neutraceutical agent. Studies in L‐carnosine had used variable dosing of 200 mg–2 g across schizophrenia and autism spectrum disorder. A recent study from Tehran proposed that this drug improves negative symptoms and cognition and highlighted that a 2 g dose of L‐Carnosine is necessary to exert any glutamatergic or NMDA modulatory effects in schizophrenia.^10^, ^11^ On the contrary, our 24‐weeks study found no change from baseline on the negative symptoms domain scores. This contrasting finding could be due to the dosing patterns (800 mg vs. 2 g), duration of study (24 vs. 8 weeks) and illness‐related variables such as chronicity, long duration of illness, and treatment resistance seen in our study population. Indian research on the optimal dose of L‐Carnosine is lacking. In the Indian setting, L‐Carnosine was commercially available as 200 mg and for study purpose the drug manufacturer had regulatory agency approval for 400 mg tablets (drug and placebo). This was the first Indian study to examine the efficacy and safety of L‐Carnosine so the study drug dosing was fixed at 400 mg for 12 weeks and increased to 800 mg till the completion of the study. Attention scores on measures of cognition were significantly improved in patients receiving 800 mg of L‐Carnosine. The US study in Schizophrenia population had used 200 mg L‐Carnosine and cited improvement in cognition but not psychopathology and also stated higher dosing as possible reasons for lack of improvement in symptoms.^10^ The other domains of cognition did not show any improvement in both arms of our study. The multiple actions of L‐carnosine in cognitive dysfunction such as oxidative stress, advanced glycation end products formation inhibition, and the glutaminergic and NMDA modulatory effects were not achieved due to insufficient dosing. Additionally, the chronic nature of illness and antipsychotic induced side effects combined may have contributed to lack of improvement in cognition except attention. The attention scores showing significant improvement at 800 mg in the active arm and the effect of placebo response are noteworthy. Therefore, a well‐designed study using optimal dosing of 1.5–2 g for longer duration can eliminate the placebo response ideally.

In summary, controlling for combined effects of confounding variables such as age and gender, matching illness severity (SANS score > 60) in the two groups, concurrent antipsychotic medications, chronic nature of the illness, and placebo response were insufficient to produce the desired results.

A larger multicenter longitudinal study using higher doses of L‐carnosine 2 g or more should be planned to explore the desired efficacy of the drug in improving all domains of cognition and negative symptoms.

This current study and all previous studies using L‐Carnosine in schizophrenia did not report any major adverse events.

## 5 LIMITATIONS AND FUTURE DIRECTION

This was a double‐blind, placebo‐controlled, prospective study with adequate sample size. However, the low dose of 400 or 800 mg has been the major limitation. A multisite study using a differential dosing of L‐Carnosine (1.5–2 g), longer follow up, utility of newer scales such as BNSS (Brief Negative Symptoms Scale) or CAINS (Clinical Assessment Interview for Negative Symptoms) and Anticholinergic burden assessment may throw more conclusions on the efficacy of the drug. Additionally, prospectively measuring serum concentrations of alanine and glutamate in an L‐Carnosine study can also establish these as putative inflammatory biomarkers in schizophrenia.

## 6 CONCLUSION

L‐Carnosine add‐on therapy (800 mg) in schizophrenia has shown improvement in executive function although negative symptoms did not improve.

## AUTHOR CONTRIBUTIONS

Study design and manuscript preparation: Hema Tharoor, Sindhu Maran. Sample Collection and assessments: Hema Tharoor, Sindhu Maran, Antra K Chandan, Manikandan Pari, Sruthi Rao. Data analysis: Jothilakshmi Durairaj. Contributed to the writing of manuscript: Hema Tharoor, Sindhu Maran.

## FUNDING INFORMATION

This study was supported by the Shield Health Care.

## CONFLICT OF INTEREST STATEMENT

There are no conflicts of interest in relation to the subject of this study.

## Supporting information


Appendix S1.

Table S1–S3.

## Data Availability

Data cannot be shared publicly because of privacy/ethical restrictions.
