# Supplementary material for: Cognitive and negative symptoms in schizophrenia with L‐Carnosine adjuvant therapy – A randomized double‐blind placebo‐controlled study
Source: Pharmacol Res Perspect. 2023 Mar 22;11(2):e01074. doi: 10.1002/prp2.1074 (PMC10031293; doi:10.1002/prp2.1074)
Supplement: Supplementary file 2 — Table S1–S3. [file PRP2-11-e01074-s002.docx]

**Table -5 Enrolment, allocation, and assessment schedule (SPIRIT format):**

| **RCT Enrolment Allocation Study Period (Post allocation)**    **Time point Visit-1 Visit -2 Visit -3 Visit- 4**  **Baseline t_1 month_  t_3 month_ t_6 month_** | | | | | |
| --- | --- | --- | --- | --- | --- |
| **ENROLMENT:**  Eligibility screen  Informed consent  Demographic data | X  X  X |  |  |  |  |
| **ALLOCATION:** |  | X |  |  |  |
| **INTERVENTIONS:**  L-Carnosine group  Placebo group |  | X  X | X  X | X  X | X  X |
| **ASSESSMENT:** |  |  |  |  |  |
| **SAPS**  **SANS**  **CGI**  **Cognitive assessment**  **Vitals and Anthropometric**  **Adverse event checklist** |  | X  X  X  X  X  X | X  X  X  X  X | X  X  X  X  X | X  X  X  X  X  X |

(RCT= Randomized Controlled Trial; SAPS= Scale for the Assessment of Positive Symptoms; SANS=Scale for the Assessment of Negative Symptoms; CGI=Clinical Global Impression.)

**Table 6- Safety monitoring schedule:**

| **Weeks/Months** | **Vital signs monitoring** | **Adverse events checklist** | **ECG** | **Complete blood count** | **LFT**  **(Liver function test)** | **RFT**  **(Renal function test)** | **RBS**  **(Random blood sugar)** |
| --- | --- | --- | --- | --- | --- | --- | --- |
| Baseline | x | x | x | x | x | x | x |
| 4 weeks | x | x |  |  |  |  |  |
| 8 weeks | x | x |  |  |  |  |  |
| 12 weeks | x | x | x | x | x | x | x |
| 16 weeks | x | x |  |  |  |  |  |
| 20 weeks | x | x |  |  |  |  |  |
| 24 weeks | x | x | x | x | x | x | x |

(ECG= Electrocardiogram; LFT = Liver Function Test; RFT= Renal Function Test; RBS =Random Blood Sugar).

**Table -7 Descriptive Characteristics of participants- Comparison using t-test and Chi-square tests:**

| **Variables** | **Group** | | |
| --- | --- | --- | --- |
|  | **Drug (n=50)** | **Placebo (n=50)** | **p Value** |
|  | **n (%)** | **n (%)** |  |
| **Gender** |  |  |  |
| Female | 15(30) | 19(38) | 0.398 |
| Male | 35(70) | 31(62) |  |
| **Marital Status** |  |  |  |
| Divorced | 1(2) | 1(2) | 0.968 |
| Married | 11(22) | 9(18) |  |
| Separated | 2(4) | 2(4) |  |
| Unmarried | 36(72) | 38(76) |  |
| **Employment Status** |  |  |  |
| Dependant | 2(4) | 2(4.2) | 0.58 |
| Employed | 10(20) | 11(22.9) |  |
| Housewife | 1(2) | 2(4.2) |  |
| Student | 0(0) | 2(4.2) |  |
| Unemployed | 37(74) | 31(64.6) |  |
|  |  |  |  |

| **Variables** | **Group** | | |
| --- | --- | --- | --- |
|  | **Drug (n=50)** | **Placebo (n=50)** | **P Value** |
|  | **Mean±SD** | **Mean±SD** |  |
| Age | 32.1±7.4 | 31±5.8 | 0.412 |
| Age of onset | 23.9±6.1 | 22.1±4.6 | 0.092 |
| Years of Education | 14.0±2.4 | 14.3±2.6 | 0.448 |
| Height Baseline | 167.9±13.8 | 165.9±13.6 | 0.569 |
| Weight Baseline | 74.8±11.1 | 77.4±16.5 | 0.476 |
| Antipsychotics dosage: |  |  |  |
| Clozapine | 90.5±60.4 | 97.8±81.0 | 0.929 |
| Amisulpride | 290.0±206.6 | 378.6±188.8 | 0.267 |
| Risperidone | 3.1±1.2 | 31.4±1.45 | 0.543 |
| Aripiprazole | 11.4±2.4 | 10.0±0 | 0.326 |
| Antipsychotics: n (%)  Clozapine  Amisulpiride  Risperidone  Aripiprazole | 19 (38)  9 (18)  18 (36)  4 (8) | 16(32)  16(32)  15(30)  3(6) | 0.452 |
| BP Systolic baseline | 114.6±13.3 | 111.4±13.2 | 0.34 |
| BP Diastolic baseline | 76±9.5 | 78.3±20.6 | 0.571 |
| Pulse baseline | 94.5±16.2 | 89.9±17.5 | 0.283 |
| Waist baseline | 99.1±11.1 | 99.2±25.5 | 0.99 |
|  |  |  |  |
|  |  |  |  |

(n=number; SD=Standard Deviation; BP=Blood Pressure)
